# Effect of changes in sleeping behavior on skeletal muscle and fat mass: a retrospective cohort study

**DOI:** 10.1186/s12889-023-16765-7

**Published:** 2023-09-28

**Authors:** Jihun Song, Sun Jae Park, Seulggie Choi, Minjung Han, Yoosun Cho, Yun Hwan Oh, Sang Min Park

**Affiliations:** 1 Department of Biomedical Sciences, Seoul National University Graduate School, Seoul, Republic of Korea; 2 Department of Internal Medicine, Seoul National University Hospital, Seoul, South Korea; 3 Department of Family Medicine, Myongji Hospital, Goyang, South Korea; 4 Total Healthcare Center, School of Medicine, Kangbuk Samsung Hospital, Sungkyunkwan University, Seoul, Republic of Korea; 5 Department of Family Medicine, Chung-Ang University Gwangmyeong Hospital, Chung-Ang University College of Medicine, Gwangmyeong, Republic of Korea; 6 Department of Family Medicine, Seoul National University Hospital, 101, Daehak-ro, Jongno- gu, Seoul, Republic of Korea

**Keywords:** Sleeping disorder, Muscle mass, Fat mass

## Abstract

**Background:**

An association between sleep behaviors and muscle-fat mass is continuously interesting topic.

**Methods:**

Based on the survey on sleep behaviors (quality and duration), the poor quality of sleep was evaluated when the subject did not feel satisfied after sleep, while the good quality was evaluated as they feel refreshed. A total of 19,770 participants were divided into the four groups according to changes in sleep quality: Good-to-Good (those who continuously maintained good quality), Good-to-Poor (those who reported initial good quality but subsequently reported a poor quality), Poor-to-Poor (those who continuously maintained poor quality), and Poor-to-Good (those who reported improved quality of sleep). As changes in skeletal muscle and fat mass index [kg/m^2^] were estimated by a validated prediction equation, multiple linear regression was used to calculate adjusted mean (adMean) of muscle and fat mass according to changes in sleep behavior.

**Results:**

When sleep duration decreased and quality of sleep deteriorated (from good to poor), fat mass index significantly increased (adMean: 0.087 for the Good-to-Good group and 0.210 for the Good-to-Poor group; *p*-value = 0.006). On the other hand, as the quality of sleep deteriorated, skeletal muscle mass more decreased despite the maintained sleep duration (adMean: -0.024 for the Good-to-Good group and − 0.049 for the Good-to-Poor group; *p*-value = 0.009).

**Conclusion:**

Our results showed that changes in sleep quality and duration affect changes in muscle and fat mass. Thus, we suggest maintaining a good quality of sleep, even if sleep duration is reduced, to preserve muscle mass and inhibit the accumulation of fat.

## Introduction

Several recommendations help secure the sleep duration, quality, and efficiency; 7–8 h sleep for recommended sleep duration for adults, limiting nap time under 30 min, and avoiding caffeine, and heavy food before sleep [1]. Despite these recommendations, however, many the middle-aged and elderly people suffer sleep disorder [2]. Sleep behavior encompasses a wide variety of factors, including subjective quality of sleep, sleep duration, time of sleep onset (time difference from time of actual sleep), awaken frequency in the middle of sleep; body activity during sleep (changes in hormone-neurotransmitter concentration, sleep-disordered breathing, and body-turning). Sleep behaviors such as sleep quality and duration are associated with health conditions such as chronic inflammation and hormonal imbalance. It has been also reported that the risk of diseases such as cardiovascular disease [3] and dementia [4, 5] increases as sleep behavior becomes worse. Some study suggested that sleep-disordered breathing and nocturnal hypoventilation may be related to the decline in cognitive function, which finally leads to dementia. Moreover, a deterioration of sleep behavior is known to worsen muscle function and cause obesity. According to a previous study, the lower muscle mass group frequently suffered poor sleep efficiency (the ratio of actual sleep time to time in bed) [6]. The persons with less sleep time have a higher fat mass index than the persons with adequate sleep [7]. The risk of general obesity, defined as body mass index (BMI) ≥ 25 kg/m^2^, in those who sleeps less than 5 h were 1.22 times higher, compared to other subjects (sleep duration: 7 h). However, there is insufficient evidence on how much muscle mass decreases and fat mass increases with respect to change in sleep duration or in quality of sleep. Most previous studies were cross-sectional studies, rather than retrospective studies. In addition, since the effect of sleep behavior on body composition is difficult to evaluate individually, more investigation regarding the interaction of factors related to sleep with body composition is required.

Herein, we have evaluated the association of changes in both sleep quality (Good or Poor) and duration on change in skeletal muscle mass and fat mass index among 19,770 people in Korea. Through our study, we hypothesized that improvement of sleep behavior would lead to the preservation of muscle mass and the reduction of fat mass in middle-aged subject.

## Methods

### Study population

Data was collected from an urban-based cohort in the Health Examinees of KoGES (HEXA) study which is conducted in the Republic of Korea. Previously, the HEXA study has been used for the examination of the influence of sleep duration and quality [8, 9]. The HEXA study was based on the cities in Korea and consisted of databases comprised of Korean adults aged between 40 and 69 years who visited study centers for health examinations for baseline (between 2004 and 2013) and base-follow up (between 2012 and 2016). A total of 27,577 participants collected from the HEXA study have participated in both health examinations at baseline and follow-up periods. Among them, 7,556 participants were excluded due to missing anthropometric data and covariates (weight, height, waist circumference, history of depression, etc.) and 251 participants also were excluded due to key variables related to sleep duration and quality. The final study population consisted of 19,770 participants.

### Assessment of sleeping behavior

Data on sleep duration and quality were collected from the results of the self-reported survey in the HEXA study. Regarding sleep duration, participants were asked: “On average, how many hours of sleep did you get per day during the past year including nap times?” Four responses were possible (under 5, 6–7, 8–9, or ≥ 10 h). Regarding sleep quality, participants were asked how often they agree with the following statement: “I don’t feel refreshed after I sleep.” Four responses were possible (always, mostly, sometimes, or not at all). The participants who were not satisfied with their sleep and responded “always” and “mostly” on the survey were considered to have poor sleep quality (the Poor group). On the other hand, the participants with responses of “sometimes” and “not at all” were considered to have good sleep quality (the Good group). Moreover, classification into the poor or good group was conducted twice during the baseline period (2004–2008) and follow-up periods (2012–2016). As a result, the study population was divided into the four groups; the Good-to-Good group (continuously maintained good quality of sleep), the Good-to-Poor group (the good sleep quality at the baseline but got poor at the follow-up periods), the Poor-to-Poor group (continuously maintained the poor quality of sleep), and the Poor-to-Good group (improved sleep quality from the baseline to the follow-up periods). Participants who maintained initial sleep quality severed as reference: the Good-to-Good and Poor-to-Poor group. In addition, all participants were classified into three groups according to the change in sleep duration: those with decreased, maintained, or increased sleep duration.

### Assessment of predicted body composition index

Using a prediction equation, appendicular skeletal muscle mass index (pAMI) and fat mass index (pFI) were assessed and calculated [10]. This prediction equation has been used previously for studies on muscle and fat mass in Korean adults [11]. To predict muscle and fat mass index, the anthropometric prediction equations were developed with multiple linear regressions and multi-variables (sex, age, height, weight, waist circumference, etc.). The correlation coefficient for variables was given from the equation. In our study, five variables (sex, age, height, weight, and waist circumference) and their correlation coefficient were used to calculate pAMI and pFI.

### Covariates: age, sex, follow-up duration, calorie, protein intake, physical activity, and others

The covariates considered were age (continuous; unit: years), sex (categorical, male or female), income level (categorical; low, middle, or high), history of depression (categorical; yes or no), follow-up duration (continuous; unit: years), BMI (continuous; unit: kg per square of the meter), calorie intake (continuous; unit: kcal per kg of weight · day), protein intake (continuous; unit: gram per kg of weight·day), fat intake (continuous; unit: gram per kg of weight·day), smoking and drinking status (categorical; never, former, or current), physical activity (categorical; yes or no). BMI was calculated by dividing the body weight (kg) by the square of height (square of the meter). Weight-standardized nutrient intake in individuals, including calorie, protein, and fat, was calculated as following: daily nutrient intake divided by the body weight. The sub-group, according to intake of protein and calorie, for stratified analyses were classified, based on the standards in men and women over the age of 40 [12].

### Statistical analysis

This retrospective cohort study was designed and conducted, based on Strengthening the Reporting of Observational Studies in Epidemiology (STROBE) guideline and its checklist.

All data collection and analysis were conducted using SAS version 9.4 (SAS Institute Inc., Cary, NC, USA). Statistical significance was defined as *p*-value < 0.05. *p*-value by chi-square test for categorical variables and analysis of variance (ANOVA) for continuous variables were used to determine the difference in descriptive characteristics. With adjusting for age, sex, physical activity, etc., multiple linear regression was conducted to determine adjusted mean and 95% confidence intervals of the change in pAMI and pFI according to the change in the sleep quality or duration.

## Results

### Characteristics of the study population

Table 1 showed the general characteristics of four groups divided according to changes in sleep quality. In the Good-to-Good, Good-to-Poor, Poor-to-Good, and Poor-to-Poor groups, 14,054, 1,629, 854, and 3,233 people participated in all surveys about changes in sleep behaviors, respectively. The percentage of 6–7 h sleep duration in each group was 66.3, 62.2, 57.1, and 60.1%, respectively, which showed a lower percentage with the poor quality of sleep. In addition, the poor quality of sleep groups suffered more depression than the good quality of sleep groups as the percentage of subjects with experience of depression was 1.4, 2.4, 5.6, and 3.4%, respectively. The weight-standardized calorie intake was 29.1 ± 9.9, 29.2 ± 11.7, 28.9 ± 11.0, 28.7 ± 10.5 kcal/kg·day in the Good-to-Good, Good-to-poor, Poor-to-Good, and Poor-to-Poor group, respectively.

**Table 1:** General characteristics of study population at baseline period

	Changes in sleep quality between baseline and follow-up periods				*p*-value
	Good-to-Good	Good-to-Poor	Poor-to-Poor	Poor-to-Good	
**Total, N (%)**	14,054 (71.1)	1,629 (8.2)	854 (4.3)	3,233 (16.4)	
**Sleep duration [h/day], N (%)**					< 0.001
≤ 5	1,443 (10.3)	228 (14.0)	191 (22.4)	496 (15.3)	
6–7	9,319 (66.3)	1,013 (62.2)	488 (57.1)	1,943 (60.1)	
8–9	3,079 (21.9)	356 (21.8)	156 (18.3)	728 (22.5)	
≥ 10	213 (1.5)	32 (2.0)	19 (2.2)	66 (2.0)	
**Age, mean ± SD [years]**	53.3 ± 8.0	53.6 ± 8.3	52.3 ± 7.6	53.1 ± 7.9	< 0.001
**Age [years], N (%)**					
40–49	4,893 (34.8)	576 (35.4)	1,142 (35.3)	333 (39.0)	
50–64	7,712 (54.9)	870 (53.4)	1,780 (55.1)	458 (53.6)	
65–80	1,449 (10.3)	183 (11.2)	311 (9.6)	63 (7.4)	
**Sex, N (%)**					< 0.001
Men	5,353 (38.1)	520 (31.9)	224 (26.2)	978 (30.2)	
Women	8,701 (61.9)	1,109 (68.1)	630 (73.8)	2,255 (69.8)	
**Income level, N (%)**					< 0.001
Low	2,547 (18.1)	360 (22.1)	213 (24.9)	801 (24.8)	
Middle	7,601 (54.1)	828 (50.8)	414 (48.5)	1,619 (50.1)	
High	3,906 (27.8)	441 (27.1)	227 (26.6)	813 (25.2)	
**History of depression, N (%)**					< 0.001
No	13,850 (98.6)	1,590 (97.6)	806 (94.4)	3,122 (96.6)	
Yes	204 (1.4)	39 (2.4)	48 (5.6)	111 (3.4)	
**Follow-up duration, mean ± SD [years]**	6.82 ± 1.72	6.62 ± 1.62	6.52 ± 1.57	7.12 ± 1.77	< 0.001
**Follow-up duration [years], N (%)**					< 0.001
5–6	5,318 (37.8)	674 (41.4)	372 (43.6)	1,000 (30.9)	
7–9	8,0323 (57.2)	892 (54.8)	461 (54.0)	2,038 (63.0)	
10–13	703 (5.0)	63 (3.9)	21 (2.5)	195 (6.0)	
**Body mass index, mean ± SD [kg/m** ^**2**^ **]**	23.9 ± 2.8	23.9 ± 2.8	23.8 ± 3.0	23.8 ± 2.8	0.561
**Body mass index [kg/m** ^**2**^ **], N (%)**					0.179
< 18.5	215 (1.5)	21 (1.3)	18 (2.1)	60 (1.9)	
18.5–25.0	9,404 (66.9)	1,065 (65.4)	558 (65.3)	2,193 (67.8)	
≥ 25.0	4,435 (31.6)	543 (33.3)	278 (32.6)	980 (30.3)	
**Nutrient intake, mean ± SD**					
**Calorie with weight [kcal/kg·day]**	29.1 ± 9.9	29.2 ± 11.7	28.9 ± 11.0	28.7 ± 10.5	0.458
**Calorie [kcal/day]**	1,779 ± 562	1,768 ± 648	1,714 ± 601	1,740 ± 628	< 0.001
**Protein with weight [g/kg·day]**	1.00 ± 0.45	0.99 ± 0.53	0.98 ± 0.50	0.97 ± 0.49	0.047
**Protein [g/day]**	61.0 ± 26.7	60.1 ± 30.0	57.7 ± 28.3	58.8 ± 29.3	< 0.001
**Fat with weight [g/kg·day]**	0.46 ± 0.30	0.46 ± 0.37	0.45 ± 0.34	0.45 ± 0.30	0.530
**Fat [g/day]**	28.2 ± 18.1	28.1 ± 21.2	26.9 ± 17.9	27.4 ± 20.3	0.044
**Drinking status, N (%)**					0.005
Never	7,367 (52.4)	874 (53.6)	443 (51.9)	1,825 (56.4)	
Former	460 (3.3)	50 (3.1)	32 (3.8)	103 (3.2)	
Current	6,227 (43.3)	705 (43.3)	379 (44.4)	1,305 (40.4)	
**Smoking status, N (%)**					< 0.001
Never	10,272 (73.1)	1,242 (76.2)	662 (77.5)	2,509 (77.6)	
Former	2,299 (16.4)	219 (13.4)	87 (10.2)	417 (12.9)	
Current	1,483 (10.6)	168 (10.3)	105 (12.3)	307 (9.5)	
**Physical activity, N (%)**					< 0.001
Low	6,024 (42.9)	759 (46.6)	474 (55.5)	1,616 (50.0)	
High	8,030 (57.1)	870 (53.4)	380 (44.5)	1,617 (50.0)	
**Fasting blood sugar, mean ± SD [mg/dL]**	94.6 ± 18.7	94.6 ± 19.6	95.0 ± 21.1	94.4 ± 19.5	0.820
**Total Cholesterol, mean ± SD [mg/dL]**	196.2 ± 34.8	196.5 ± 33.4	197.6 ± 35.3	196.1 ± 35.2	0.701

Study population was divided into 4 groups according to sleeping disorder between baseline and follow-up: Good-to-Good, Good-to-Poor, Poor-to-Good, and Poor-to-Poor.

*p*-values were calculated using the chi-squared test for categorical variables and analysis of variance for continuous variables

Abbreviation: N (number of people); SD (standard deviation)

### Changes in muscle and fat mass according to changes in both sleep duration and quality

**Fig. 1:**
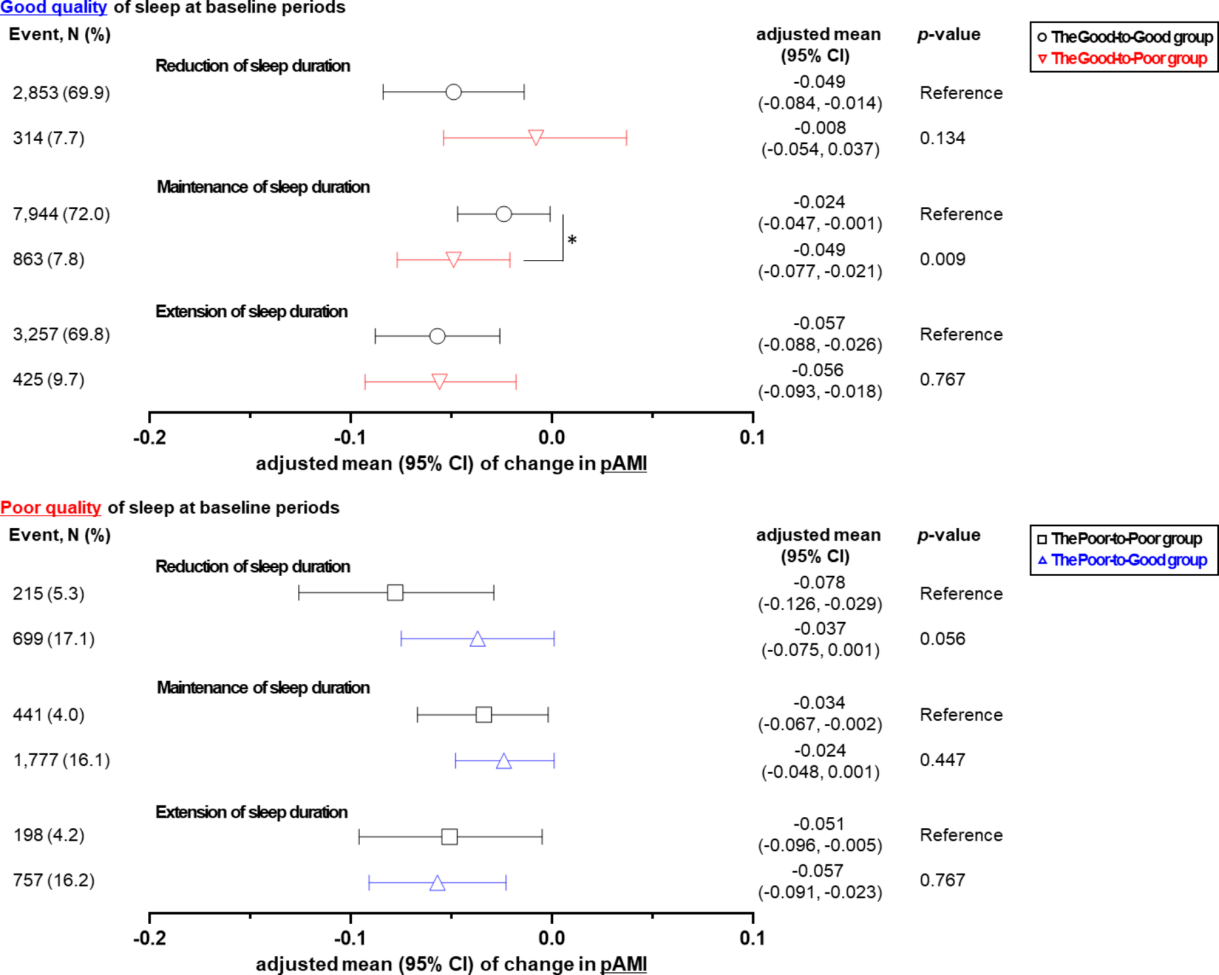
Adjusted mean of change in muscle mass index according to change in sleep duration (reduction, maintenance, or extension) and quality (good or poor at baseline periods and good or poor at follow-up periods). Adjusted means (95% confidence interval (CI)) of change in predicted appendicular skeletal muscle mass index (pAMI). Adjusted mean were calculated using linear regression analysis after adjusting for the sleeping duration at baseline, age, sex, income level, history of depression, calorie intake, protein intake, fat intake, body mass index, follow-up duration, drinking status, smoking status, and physical activity. *p-value < 0.05

**Fig. 2:**
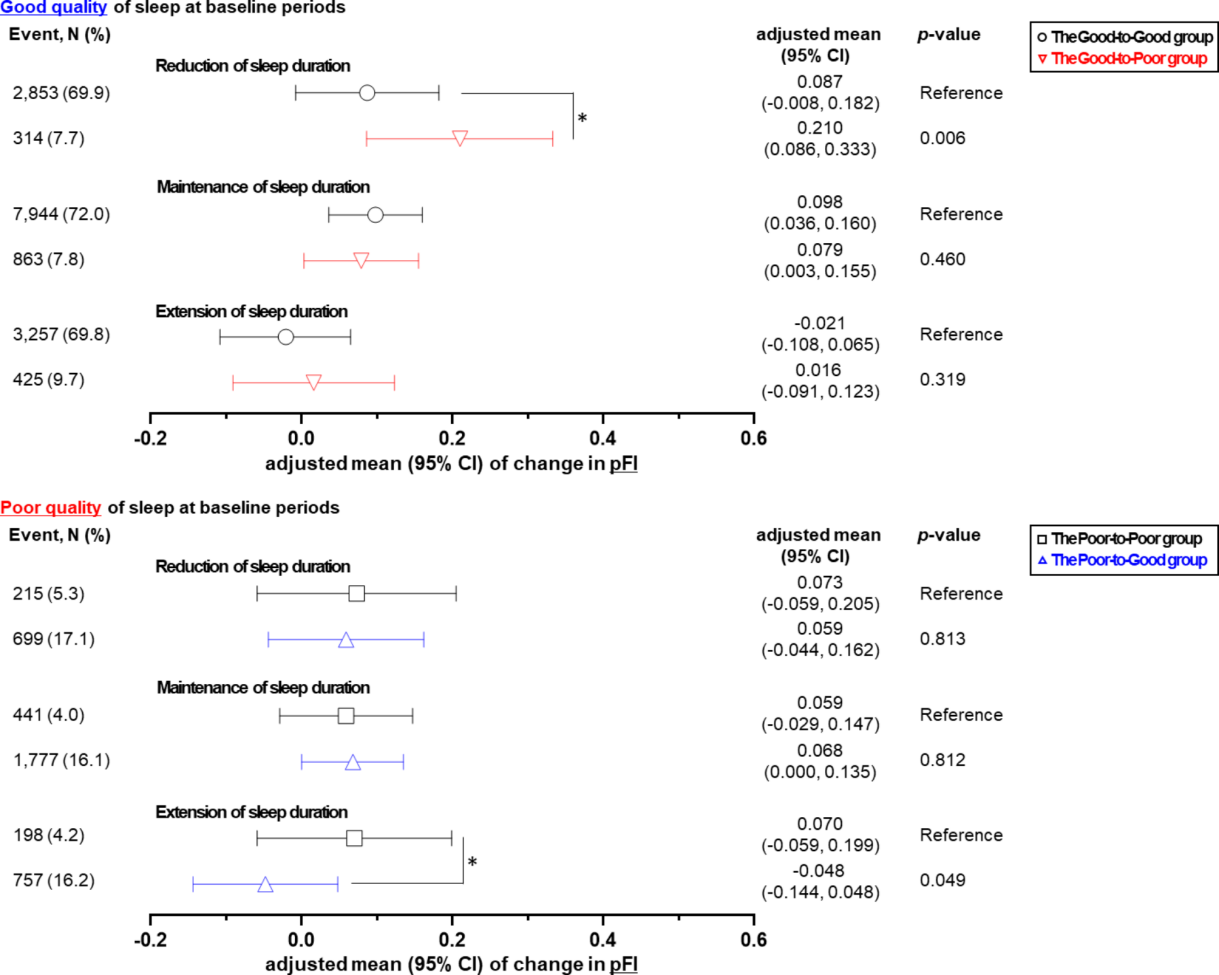
Adjusted means of change in fat mass index according to change in sleep duration (reduction, maintenance, or extension) and quality (good or poor at baseline periods and good or poor at follow-up periods). Adjusted means (95% CI) of change in predicted fat mass index (pFI). Adjusted means were calculated using linear regression analysis after adjusting for the sleeping duration at baseline, age, sex, income level, history of depression, calorie intake, protein intake, fat intake, body mass index, follow-up duration, drinking status, smoking status, and physical activity. *p-value < 0.05

Association between change in sleep quality and body composition was revealed by stratified analysis according to the reduction, maintenance, and extension of sleep duration as shown in Figs. 1 and 2. For those with poor quality of sleep at baseline, when sleep time was reduced, the adjusted mean of pAMI in the Poor-to-Poor and Poor-to-Good groups increased from − 0.078 to -0.037 (*p*-value = 0.056) as shown in Fig. 1. In other words, degree of muscle reduction was higher in the poor-to-poor group compared to those in the poor-to-good. For those with good quality of sleep at baseline, when sleep time was maintained, the adjusted mean of pAMI decreased: -0.024 in the Good-to-Good group and − 0.049 in the Good-to-Poor group (*p*-value = 0.009). With maintained sleep duration, the reduction of pAMI was mitigated: -0.034 in the Poor-to-Poor group and − 0.024 in the Poor-to-Good group (*p*-value = 0.447).

On the other hand, when the quality of sleep deteriorated in the initial good quality of sleep at reduced sleep duration, the adjusted mean of pFI increased significantly from 0.087 to 0.210 (*p*-value = 0.006) as shown in Fig. 2. When the quality of sleep improved and sleep duration increased, the adjusted mean of pFI in the Poor-to-Poor and Poor-to-Good groups decreased from 0.070 to -0.048 (*p*-value = 0.049).

The decrease in pAMI was alleviated regardless of age or sex when the quality of sleep improved and sleep duration was reduced (Table 2). The results of stratified analysis in even low physical activity showed that the improved sleep quality from poor to good alleviated the decrease of pAMI: -0.065 in the Poor-to-Poor group and − 0.046 in the Poor-to-Good group (*p*-value = 0.183). When the quality of sleep deteriorated in the low calorie intake group, the adjusted mean of pFI significantly increased: 0.036 in the Good-to-Good group and 0.089 in the Good-to-Poor group (*p*-value = 0.021).

**Table 2:** Stratified analyses of change in body composition according to behaviors of sleep between baseline and follow-up period in the groups who reduced sleep duration

	Dissatisfaction with sleep quality among participants who reduced sleep duration					
	Good quality at baseline period		*p*-value	Poor quality at baseline period		*p*-value
	Good-to-Good	Good-to-Poor		Poor-to-Poor	Poor-to-Good	
	**Change in pAMI, adjusted means (95% CI) [kg/m** ^**2**^ **]**					
**Age [year]**						
40–49	0.001 (-0.029, 0.031)	-0.021 (-0.057, 0.015)	0.052	0.000 (-0.039, 0.039)	0.002 (-0.030, 0.034)	0.882
50–64	-0.049 (-0.07, -0.028)	-0.050 (-0.077, -0.024)	0.905	-0.068 (-0.099, -0.037)	-0.046 (-0.069, -0.023)	0.112
65–80	-0.135 (-0.195, -0.076)	-0.103 (-0.173, -0.032)	0.145	-0.135 (-0.224, -0.047)	-0.128 (-0.192, -0.063)	0.847
**Sex**						
Men	-0.101 (-0.145, -0.057)	-0.111 (-0.162, -0.061)	0.493	-0.098 (-0.157, -0.038)	-0.097 (-0.144, -0.050)	0.990
Women	-0.013 (-0.037, 0.010)	-0.014 (-0.041, 0.012)	0.892	-0.029 (-0.058, -0.001)	-0.011 (-0.036, 0.014)	0.085
**BMI [kg/m** ^**2**^ **]**						
< 18.5	0.109 (0.004, 0.214)	0.204 (0.060, 0.348)	0.081	0.153 (0.011, 0.294)	0.193 (0.077, 0.309)	0.536
18.5–25.0	-0.014 (-0.032, 0.004)	-0.021 (-0.044, 0.002)	0.360	-0.030 (-0.056, -0.004)	-0.014 (-0.034, 0.006)	0.176
≥ 25.0	-0.093 (-0.126, -0.059)	-0.095 (-0.136, -0.054)	0.862	-0.099 (-0.147, -0.052)	-0.090 (-0.127, -0.053)	0.658
**Protein intake [g/day]**						
Low (< 40)	-0.028 (-0.062, 0.005)	-0.028 (-0.071, 0.015)	0.976	-0.038 (-0.086, 0.009)	-0.026 (-0.062, 0.011)	0.548
Middle (40–80)	-0.040 (-0.061, -0.02)	-0.043 (-0.068, -0.018)	0.746	-0.053 (-0.081, -0.024)	-0.035 (-0.057, -0.013)	0.173
High (≥ 80)	-0.043 (-0.089, 0.003)	-0.062 (-0.119, -0.005)	0.298	-0.057 (-0.123, 0.01)	-0.052 (-0.102, -0.002)	0.864
**Physical activity**						
Low	-0.052 (-0.077, -0.027)	-0.043 (-0.074, -0.013)	0.413	-0.065 (-0.098, -0.032)	-0.046 (-0.073, -0.020)	0.183
High	-0.032 (-0.053, -0.010)	-0.047 (-0.074, -0.020)	0.108	-0.039 (-0.072, -0.007)	-0.031 (-0.055, -0.007)	0.584
	**Change in pFI, adjusted means (95% CI) [kg/m** ^**2**^ **]**					
**Age [year]**						
40–49	0.184 (0.099, 0.269)	0.157 (0.056, 0.258)	0.402	0.228 (0.117, 0.339)	0.152 (0.062, 0.243)	0.102
50–64	0.007 (-0.049, 0.064)	0.039 (-0.033, 0.111)	0.213	-0.026 (-0.110, 0.057)	-0.022 (-0.084, 0.041)	0.906
65–80	-0.060 (-0.215, 0.096)	0.063 (-0.120, 0.247)	0.035*	-0.120 (-0.351, 0.111)	-0.084 (-0.252, 0.084)	0.727
**Sex**						
Men	-0.034 (-0.124, 0.056)	0.000 (-0.104, 0.104)	0.260	0.029 (-0.094, 0.151)	-0.083 (-0.180, 0.013)	0.021*
Women	0.081 (0.001, 0.160)	0.103 (0.013, 0.192)	0.374	0.053 (-0.042, 0.148)	0.059 (-0.024, 0.142)	0.857
**BMI [kg/m** ^**2**^ **]**						
< 18.5	0.428 (0.130, 0.725)	0.701 (0.291, 1.110)	0.077	0.541 (0.140, 0.942)	0.487 (0.158, 0.817)	0.770
18.5–25.0	0.124 (0.073, 0.176)	0.154 (0.090, 0.219)	0.187	0.105 (0.031, 0.179)	0.088 (0.031, 0.144)	0.604
≥ 25.0	-0.074 (-0.162, 0.015)	-0.067 (-0.175, 0.041)	0.858	-0.051 (-0.178, 0.075)	-0.098 (-0.195, -0.001)	0.397
**Calorie intake** ^**a**^ **[kcal/day]**						
Low	0.036 (-0.017, 0.088)	0.089 (0.024, 0.155)	0.021*	0.049 (-0.028, 0.125)	0.006 (-0.052, 0.064)	0.217
Middle	0.116 (0.019, 0.213)	0.075 (-0.043, 0.192)	0.266	0.096 (-0.035, 0.226)	0.090 (-0.014, 0.195)	0.921
High	0.086 (-0.107, 0.278)	0.090 (-0.142, 0.321)	0.956	0.004 (-0.256, 0.264)	0.046 (-0.158, 0.251)	0.690
**Physical activity**						
Low	0.037 (-0.033, 0.106)	0.069 (-0.015, 0.154)	0.255	0.010 (-0.082, 0.102)	0.013 (-0.061, 0.087)	0.938
High	0.073 (0.015, 0.130)	0.089 (0.016, 0.161)	0.538	0.100 (0.012, 0.189)	0.038 (-0.026, 0.102)	0.126

adjusted means were calculated using linear regression analysis after adjusting for the sleeping duration at baseline, age, sex, income level, history of depression, calorie intake, protein intake, fat intake, BMI, follow-up duration, drinking status, smoking status, and physical activity

^a^Men: Low (< 2,400); middle (2,400 ≤, < 3,100); high (≤ 3,100); Women: Low (< 1,700); middle (1,700 ≤, < 2,400); high (≤ 2,400)

Abbreviation: pAMI (predicted appendicular skeletal muscle mass index); pFI (predicted fat mass index); BMI (body mass index); CI (confidence interval)

*p-value < 0.05

## Discussion

In 19,770 participants, appendicular skeletal muscle mass decreases and fat mass increases in case of the poor quality and reduced duration of sleep. Although the sleep duration decreased, the decrease in muscle mass was alleviated as the quality of sleep improved. The poor quality of sleep further reduced muscle mass although sleep duration was maintained. Amount of accumulation of fat mass increased as the sleep duration decreased and quality deteriorated at the same time.

Previously, it has been reported that body composition is affected by factors related to sleep including quality [13], duration [14, 15], and efficiency (e.g. time spent in bed at night over actual sleep duration) [6]. Those with adequate sleep duration had less fat mass index than those belonging to the lower sleep duration group [7]. Thus, reduced sleep duration might be a modifiable risk factor for general obesity. Also, a more frequent ratio of low muscle mass to normal was reported in the reduced sleep duration group than the adequate sleep duration group [16].

Pathophysiological mechanism between sleep quality and muscle mass or fat mass is not fully understood. Hormone secretion control, immune response, oxidative stress, and biorhythms, however, have been considered related-factors between sleep health and phenotypes of body composition. First, results in this study might be caused by the effects of several hormones (Insulin-like Growth Factor 1 (IGF-1), testosterone, etc.) controlled by the quality and duration of sleep [17, 18]. The amount of secreted IGF-1 was rapidly reduced in the sleep-deprived group of rats [17]. Because IGF-1-mediated signaling stimulates the protein synthesis of muscle, skeletal muscle mass might be regulated by sleep duration [19]. In addition, inflammation caused by poor sleep quality might adversely affect muscle regeneration and growth and increase fat mass. The level of secreted cytokines such as Interleukin 6 (IL-6) and tumor necrosis factor-alpha (TNF-α) in the poor sleep quality group whose sleep was interrupted was significantly higher compared to that of the good quality group (uninterrupted sleep). In the sleep-deprived participants, the relative mRNA expression of IL-6 and TNF-a increased by 3-fold and 2-fold, respectively. Excessive secreted cytokines and inflammation adversely affect muscle regeneration [20]. Moreover, the poor quality of sleep further worsens inflammatory response and obesity state since chronic inflammation frequently occurs in the obese person [21]. The poor quality of sleep usually includes the abnormal reduction in respiratory volume and frequency, known as sleep apnea [22]. As sleep apnea causes oxidative stress, an increase in fat mass would occur [23, 24]. As the sleep apnea causes a large number of reactive oxygen species (ROSs), ROSs lead to fat accumulation and obesity, because of an excess supply of energy substrates with mitochondrial dysfunction and ROS signaling. Finally, sleep quality would not directly affect muscle and fat mass but through an intermediary: a biorhythm including cardiac rhythm and a regular mealtime [25]. A previous study showed that circadian clock-regulated activity levels may have an important role in the growth of skeletal muscle [26]. Since most biorhythms are adjusted according to physical activity and exposure to sunlight, sleep behavior could control these rhythms. Thus, the decrease in skeletal muscle mass when sleep duration is reduced and sleep quality is poor might interrupt cardiac rhythm.

In our study, changes in the quality and duration of sleep affected change in body composition in an interdependent manner. Previous studies have tended to focus on only the independent effect of each sleep-related factor. However, some exceptions must be considered: the increase in sleep duration does not necessarily guarantee a good quality of sleep and proper sleep duration is not achieved even if participants are satisfied with sleep quality. To confirm the relationship between factors of sleep and body composition, the effect of changes in the sleep-related factors was analyzed in a large population after multiple variable covariates (calorie, protein, and fat intake). Not only did we analyze the effect of sleep quality on muscle mass at a single point in time, but we also demonstrated that continuous good sleep quality could help to maintain muscle mass, despite reduced sleep duration.

Despite these strengths, our study contains certain limitations. First, the predicted muscle and fat mass index, derived from anthropometrical and sociodemographic variables, has the difference from the directly measured or actual values. Thus, additional study based on the measured value and comparison of those results are necessary, although the coefficient of determination (R^2^) of prediction equation was generally 70% with evaluating the accuracy of the measured and predicted one. Furthermore, all factors related to sleep in this study are based on self-report. Because of the temporary psychological and physical state before the survey, the responses might not represent the overall sleep behavior of the subject. There is a possibility that some bias would disturb our result. For example, longer nap times may have affected the quality of sleep. Even if the absolute sleep duration is long, longer nap times might decrease the overall quality of sleep. Therefore, by reducing nap times, and hence the overall sleep duration, we may observe a restored quality of sleep as well as improvement in other factors, such as chronic inflammation, hormonal imbalance, muscle, and fat mass.

## Conclusion

Both changes in sleep duration and changes in sleep quality affect the degree of muscle mass reduction and the accumulation of fat. Although sleep duration was maintained, muscle mass more decreases as the quality of sleep became poor. In addition, continuously maintaining a good quality of sleep prevented an increase in the amount of fat even when the sleep duration decreased. Thus, we suggested that maintaining a good quality of sleep, even if sleep duration is reduced, would preserve muscle mass and inhibit the accumulation of fat.

## Data Availability

The original data in this study were provided by Korea Disease Control and Prevention Agency (KDCA) with permission. The processed data and SAS-code will be shared on request to the corresponding author after permission of co-authors and KDCA.
